# Supplementary material for: A rare oasis effect for forage fauna in oceanic eddies at the global scale
Source: Nat Commun. 2024 Jun 6;15:4834. doi: 10.1038/s41467-024-49113-3 (PMC11156932; doi:10.1038/s41467-024-49113-3)
Supplement: Supplementary file 1 — Supplementary Information [file 41467_2024_49113_MOESM1_ESM.pdf]

SUPPLEMENTARY MATERIAL of

A rare oasis effect for forage fauna in oceanic eddies at the global scale

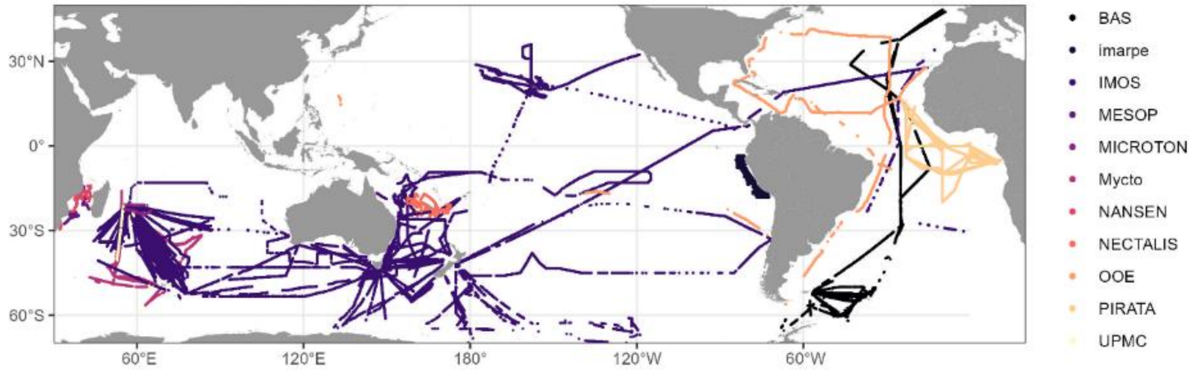

Figure S1. Map of all available acoustic data colored by data source.

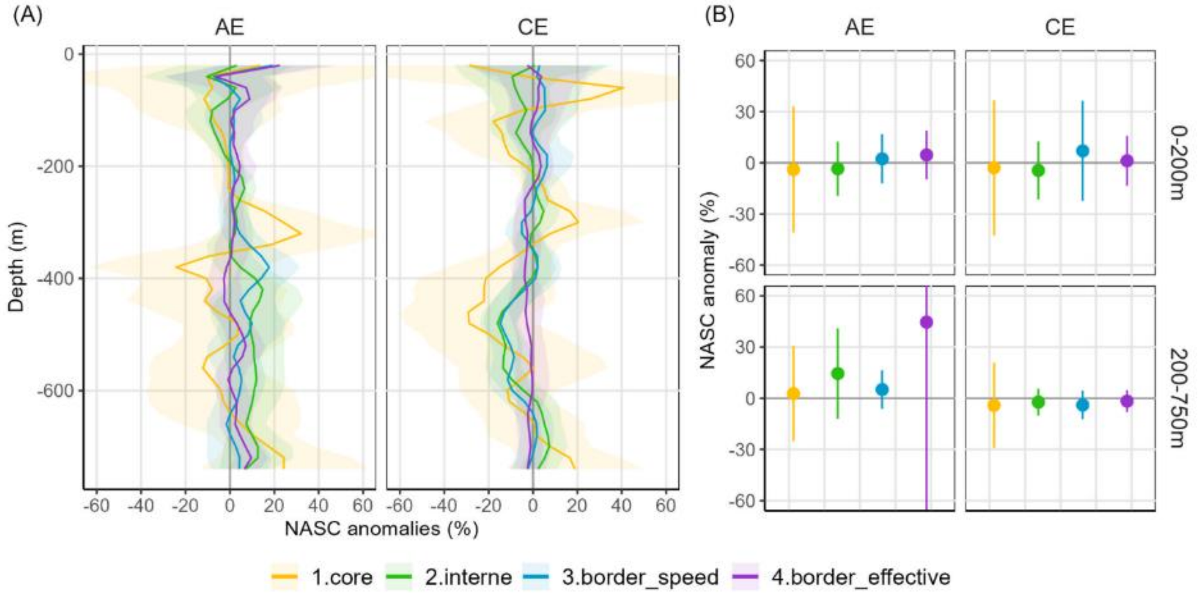

Figure S2. NASC anomalies by eddy regions with (A) vertical profiles and (B) mean value by vertical layer (*rows*) for AE and CE (*columns*). Vertical ribbons (A) and vertical bars (B) show the 95% confidence interval (1.96*standard deviation/root square of number of observations) of the mean values.

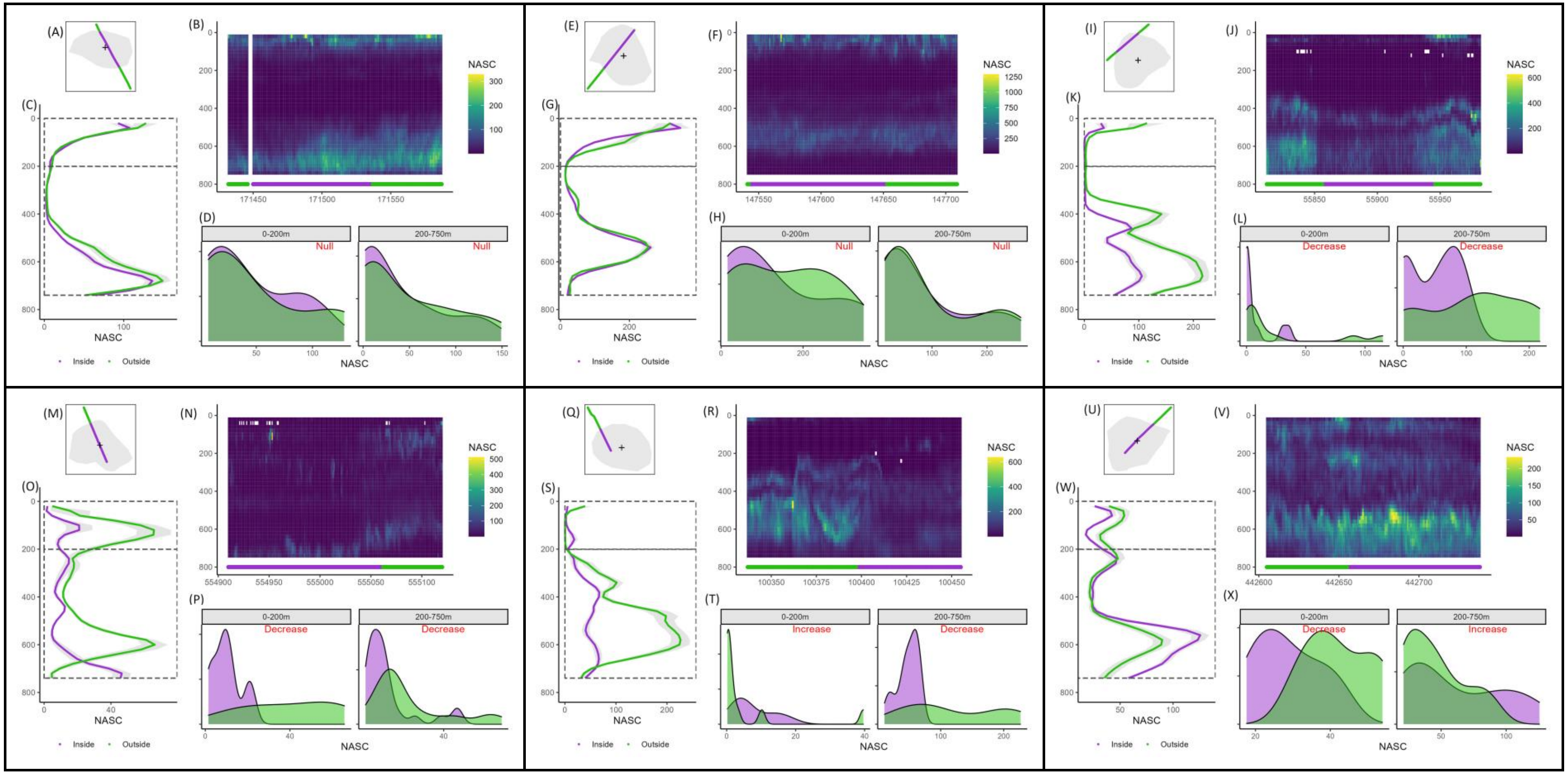

Figure S3. 6 examples of sampled eddies. In each Figure, there are (A, E, I, M, Q, U) the acoustic track (colored line) crossing the eddy (gray polygon); (B, F, J, N, R, V) the corresponding echogram, with the purple-green line at the bottom corresponding to the inside(purple)-outside(green) points to help the reader to locate the echogram in the eddy. (C, G, K, O, S, W) The mean NASC vertical profiles inside (purple) and outside (green) the eddy. The dotted rectangles show the two vertical layers considered used on the density panels. (D, H, L, P, T, X) The distributions of NASC values outside (green) and inside (purple) in the two vertical layers (*columns*). The red texts give the result of the Wilcoxon test for each case (lower [i.e. decrease effect] and higher distribution [i.e. increase effect] of the inside compared to the outside distribution, and Null a non-significant difference between the two distributions).

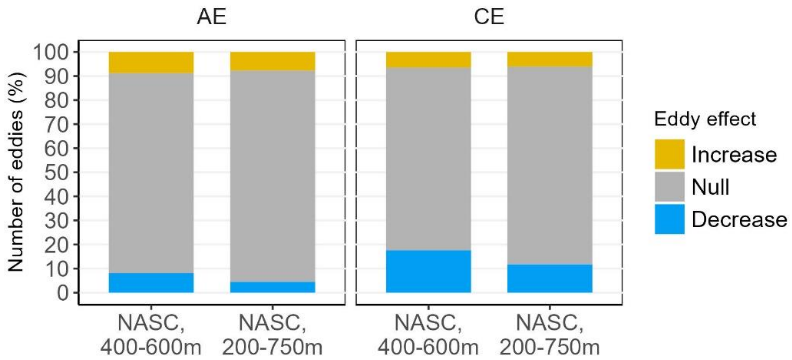

Figure S4. Eddy percentage (based on a Wilcoxon distribution test at 95% confidence level) significantly influencing (*yellow* and *blue*) or not (*grey*) forage fauna density in two vertical layers (*x-axis*) for AE and CE (*columns*).

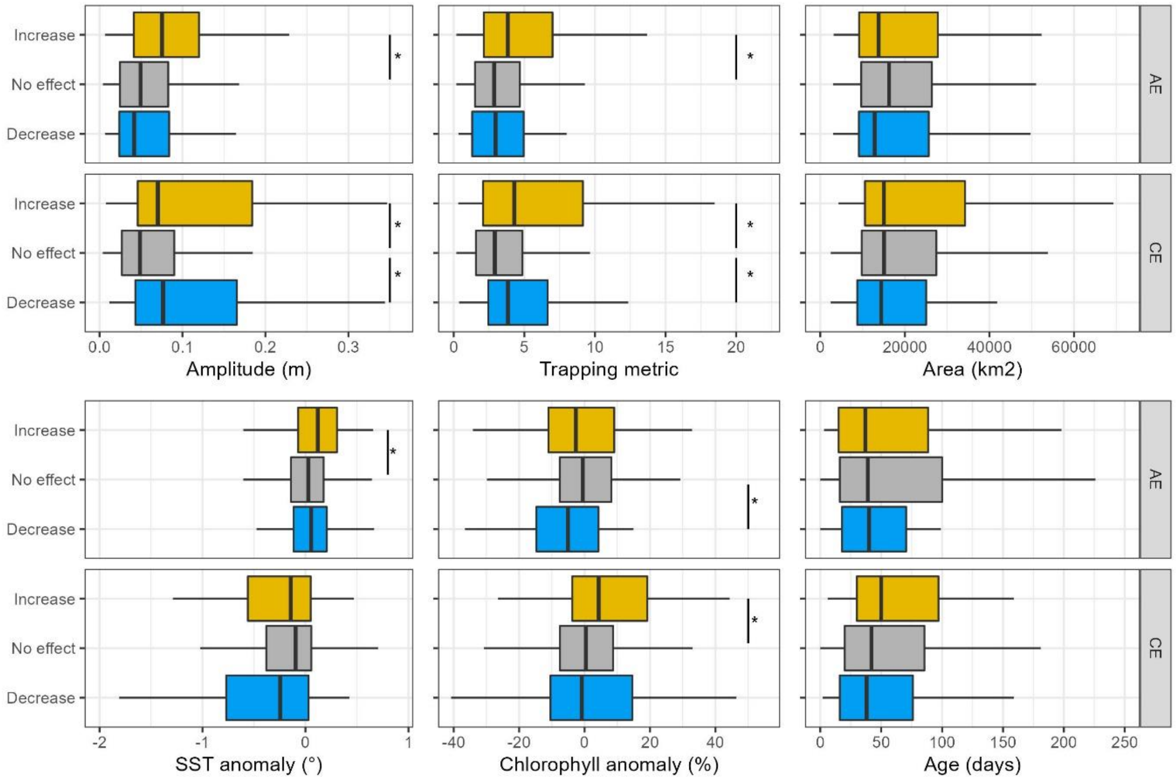

Figure S5. Distributions of the 6 eddy characteristics (amplitude, trapping ability, area, SST eddy signal, chlorophyll eddy signal, and lifespan) in the eddies increasing (*yellow*), decreasing (*blue*) and not-affecting (*gray*) the forage fauna in Anticyclonic (AE) and Cyclonic (CE) eddies (*rows*). * are written for the characteristics with a significant (two-sided Wilcoxon test with a 5% level) difference between the affecting and not-affecting distributions. n=62 increasing, n=65 decreasing and n=864 null-effect independent eddies were tested in the Wilcoxon tests. The boxplot bounds are the 0.25 (Q25) and 0.75 (Q75) quantiles, the inside vertical line shows the median, and the horizontal segments are the minimal value [Q25 - 1.5*(Q75-Q25)], and the maximal value [Q3 + 1.5*(Q75-Q25)].

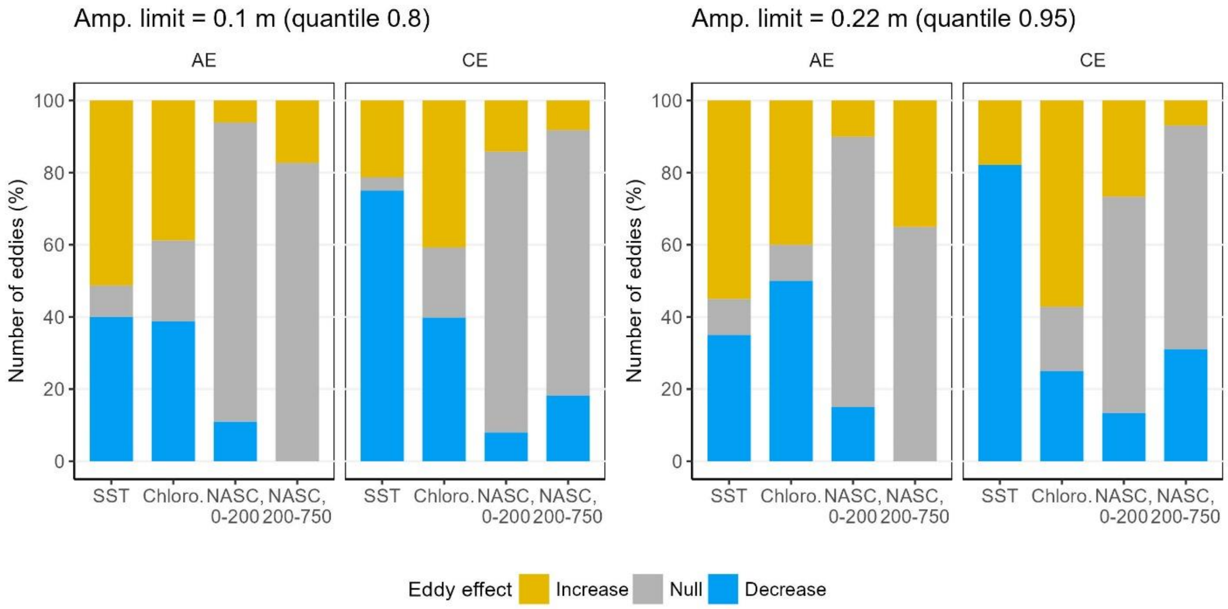

Figure S6. Eddy percentage significantly influencing (two-sided Wilcoxon test with a 5% level) (*yellow* and *blue*) or not (*gray*) SST, chlorophyll and forage fauna density in two vertical layers (*x-axis*) for AE and CE (*columns*) for the strongest eddies, i.e. with an amplitude higher 0.1 m (left) and 0.22 m (right).

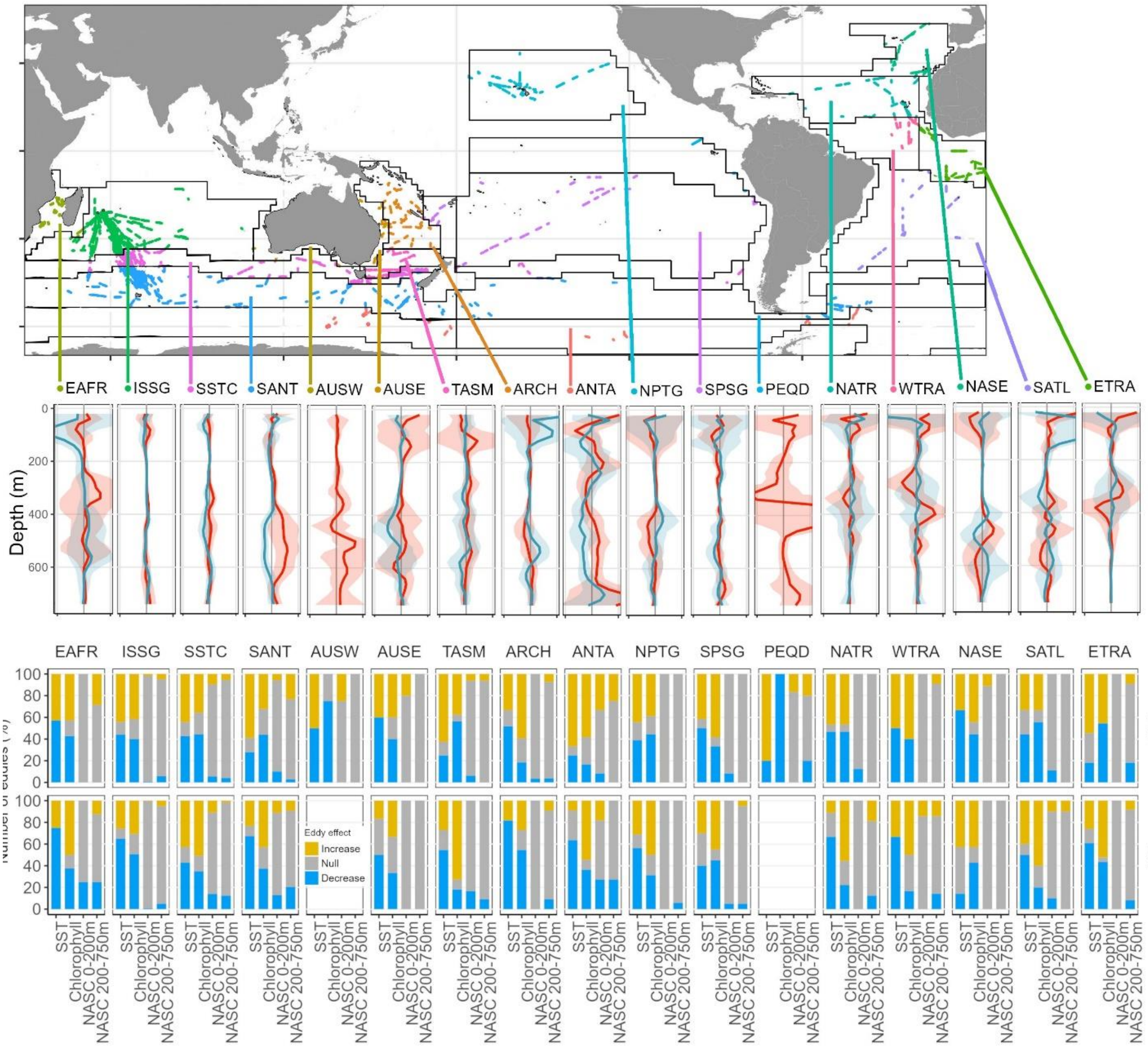

Figure S7. (**Top**) Map with the limit of the Longhurst regions; (M**iddle**) Acoustic backscatter anomalies across depth for AE (*red*) and CE (*blue*) in each sampled Longhurst regions. Vertical ribbons show the 95% confidence interval (1.96*standard deviation/root square of number of observations) of the mean values. (**Bottom**) Eddy percentage significantly influencing (*yellow* and *blue*) or not (*gray*) SST, chlorophyll and forage fauna density in two vertical layers (*x-axis*) for AE and CE (*rows*) in each sampled Longhurst regions. EAFR: Eastern African coast; ISSG: Indian South subtropical gyre; SSTC: South subtropical convergence; SANT: Sub Antarctic water ring; AUSW: Western Australian and Indonesian coast; AUSE: East Australian coast; TASM: Tasman Sea; ARCH: Archipelagic deep Basins; ANTA: Antarctic; NPTG: North Pacific Tropical gyre; SPSG: South Pacific gyre; PEQD: Pacific equatorial divergence; NATR: North Atlantic tropical gyre; WTRA: Western tropical Atlantic; NASE: Northeast Atlantic subtropical gyre; SATL: South Atlantic gyre; ETRA: Eastern tropical Atlantic.

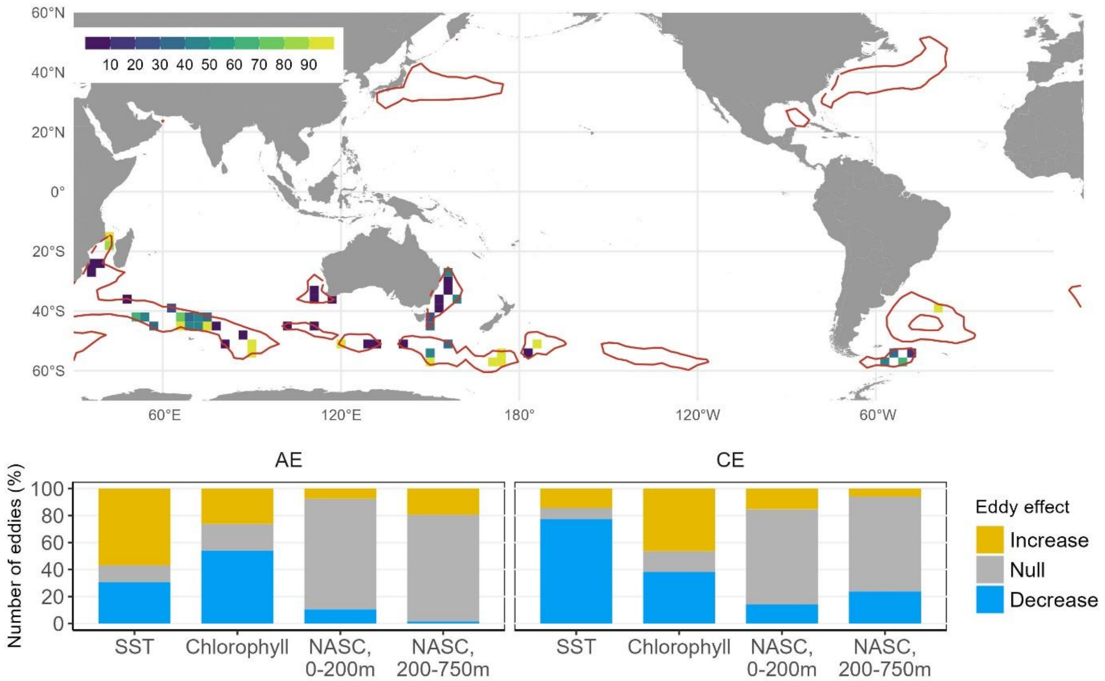

Figure S8. (*Top*) Map showing the percentage of affecting eddies (*colors*), compared to not-affecting eddies on a 3° spatial grid for spatial cell with a mean eddy amplitude superior or equal to 0.1m. (*Bottom*) Eddy percentage in these high mean eddy amplitude regions significantly influencing (*yellow* and *blue*) or not (*gray*) SST, chlorophyll and forage fauna density in two vertical layers (*x-axis*) for AE and CE (*columns*). Red contours show the regions with eddy mean amplitude higher than 0.1 m.

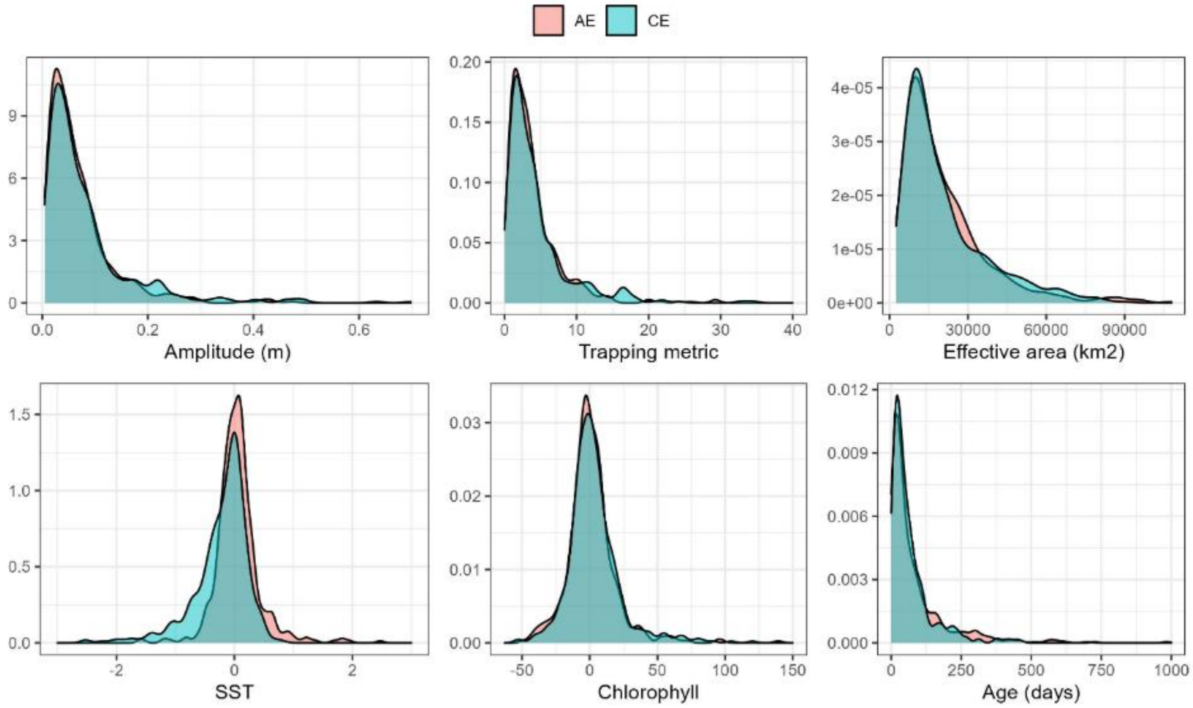

Figure S9. Distributions of eddy characteristic values in AE and CE sampled in the study (*colors*).

Table. S1. Overview of eddy influence on forage fauna from most relevant previous paper.

Reference	First author and date (for Fig. S6)	Number of citations	Number of sampled eddy	Region	Forage fauna sampling	Main result	Process tested	Process discussed
1	Annasawmy 2020	18	2 (1 AE, 1 CE)	Mozambique Channel	Acoustic	Enhancement in CE	-	Enhancement of primary production and zooplankton
2	Behagle 2014	44	8 (4 AE, 4 CE)	Mozambique Channel	Acoustic	Not consistent across surveys (enhancement in CE for 2 surveys, and in AE for 1 survey)	-	Age of eddies, the location of the creation, the trajectory and history. Strongest AE sampled which is enhanced. Proximity to the continental shelf. Mobility of organisms.
3	Brandt 1983	27	7 (AE)	Eastern Australia	Trawling	Different communities inside the eddies	-	Degree of the eddies' physical isolation after separation from the East Australian Current and season.
4	Della Penna 2020	23	13 (7 CE, 6 AE)	Northwest Atlantic	Acoustic	Enhancement in AE	Positive relationship between amplitude and acoustic anomaly	Warm temperature (positively influence metabolic rates, so growth and repro).
5	Della Penna 2022	1	1 (CE)	Southern Ocean	Acoustic	Decrease in CE	-	Trapping of eddies acoustic signal, for a 27-days period.
6	Devine 2021	4	2 (AE)	Northwest Atlantic	Trawling	Higher diversity inside AE and different communities	-	Swimming capability
7	Drazen 2011	78	1 (CE)	Hawaiian	Trawlin	Enhancement in CE	-	Possible enhancement of

				waters	g			zooplankton
8	Fennell 2015	4	? (three transects)	North Atlantic	Acoustic	Enhancement in region with AE	-	Advective effect (current)
9	Godo 2012	191	4 (AE)	North Atlantic	Acoustic and trawling	Enhancement in AE	-	passive transport and attracted by zooplankton
10	Griffiths 1986	37	1 (AE)	Eastern Australia	Trawlin g	Different communities inside the eddy	-	Tapping of communities
11	Kwong 2020	13	3 (2 CE, 1 AE)	Eastern Australia	Trawlin g	No difference between CE and AE	-	Bottom-up effect
12	Perelman 2023	0	?	Eastern Pacific	Acoustic	Enhancement in CE	-	Enhancement of primary production
13	Potier 2014	38	?	Mozambiqu e Channel	Trawlin g	No eddy effect	-	Source of the eddy water
14	Sabarros 2009	88	7 (?)	Mozambiqu e Channel	Acoustic	Enhancement in both AE and CE (at the edge)	-	-
15	Wang 2022	6	1 (AE)	Northwest Pacific	Acoustic	Enhancement in AE (in the core)	Warm temperature and rich in oxygen	Energy conservation and predator avoidance (at day). Convergence effect (at nigh)
16	Zhang 2022	0	5 (3 AE, 2 CE)	Northwest Pacific		Enhancement in AE (one species)	Temperature and primary production	Warm temperature optimum

Table. S2. Sources of acoustic data. Creator, repository details, research program, and main surveyed area (Table reproduced from[17]).

Creator	Repository	Program	Area	References
CSIRO, Australia	AODN	IMOS SOOP-BA	South Indian	18,19
IRD, France	SEANOE	NANSEM	Mozambique channel	21
IRD, France	SEANOE	MICROTON	Mozambique channel	21
IRD, France	SEANOE	MESOP	Mozambique channel	21
IRD, France	SEANOE	PIRATA	Eastern tropical Atlantic	20,21
BAS, United Kingdom	BODC	AMT, WCB	Atlantic	22,23
IMARPE, Peru	PANGAEA	Survey 0110-11	Peruvian Humboldt Current System	24,25
UPMC, France	MESOPP	Themisto	South Indian	26,27
CSIC, Spain	PANGAEA	Malaspina	Circumnavigation 40°N-40°S	24,28
Institute of Marine Research	IMR	One Ocean Expedition	Global	https://ftp.nmdc.no/nmdc/IMR/OneOcean

References

1. Annasawmy, P. *et al.* Micronekton distribution as influenced by mesoscale eddies, Madagascar shelf and shallow seamounts in the south-western Indian Ocean: An acoustic approach. *Deep Sea Res. Part II Top. Stud. Oceanogr.* 104812 (2020) doi:10.1016/j.dsr2.2020.104812.
2. Behagle, N. *et al.* Mesoscale features and micronekton in the Mozambique Channel: An acoustic approach. *Deep Sea Res. Part II Top. Stud. Oceanogr.* **100**, 164–173 (2014).
3. Brandt, S. B. Temporal and spatial patterns of lanternfish (family Myctophidae) communities associated with a warm-core eddy. *Mar. Biol.* **74**, 231–244 (1983).

4. Della Penna, A. & Gaube, P. Mesoscale Eddies Structure Mesopelagic Communities. *Front. Mar. Sci.* **7**, (2020).
5. Della Penna, A. *et al.* The Impact of a Southern Ocean Cyclonic Eddy on Mesopelagic Micronekton. *J. Geophys. Res. Oceans* **127**, (2022).
6. Devine, B., Fennell, S., Themelis, D. & Fisher, J. A. D. Influence of anticyclonic, warm-core eddies on mesopelagic fish assemblages in the Northwest Atlantic Ocean. *Deep Sea Res. Part Oceanogr. Res. Pap.* **173**, 103555 (2021).
7. Drazen, J. C., De Forest, L. G. & Domokos, R. Micronekton abundance and biomass in Hawaiian waters as influenced by seamounts, eddies, and the moon. *Deep Sea Res. Part Oceanogr. Res. Pap.* **58**, 557–566 (2011).
8. Fennell, S. & Rose, G. Oceanographic influences on Deep Scattering Layers across the North Atlantic. *Deep Sea Res. Part Oceanogr. Res. Pap.* **105**, 132–141 (2015).
9. Godo, O. R. *et al.* Mesoscale Eddies Are Oases for Higher Trophic Marine Life. *PLOS ONE* **7**, e30161 (2012).
10. Griffiths, F. B. & Wadley, V. A. A synoptic comparison of fishes and crustaceans from a warm-core eddy, the East Australian Current, the Coral Sea and the Tasman Sea. *Deep Sea Res. Part Oceanogr. Res. Pap.* **33**, 1907–1922 (1986).
11. Kwong, L. E., Henschke, N., Pakhomov, E. A., Everett, J. D. & Suthers, I. M. Mesozooplankton and Micronekton Active Carbon Transport in Contrasting Eddies. *Front. Mar. Sci.* **6**, (2020).
12. Perelman, J. N., Ladroit, Y., Escobar-Flores, P., Firing, E. & Drazen, J. C. Eddies and fronts influence pelagic communities across the eastern Pacific Ocean. *Prog. Oceanogr.* 102967 (2023) doi:10.1016/j.pocean.2023.102967.

13. Potier, M., Bach, P., Ménard, F. & Marsac, F. Influence of mesoscale features on micronekton and large pelagic fish communities in the Mozambique Channel. *Deep Sea Res. Part II Top. Stud. Oceanogr.* **100**, 184–199 (2014).
14. Sabarros, P., Ménard, F., Lévénez, J., Tew-Kai, E. & Ternon, J. Mesoscale eddies influence distribution and aggregation patterns of micronekton in the Mozambique Channel. *Mar. Ecol. Prog. Ser.* **395**, 101–107 (2009).
15. Wang, Y., Zhang, J., Yu, J., Wu, Q. & Sun, D. Anticyclonic mesoscale eddy induces mesopelagic biomass hotspot in the oligotrophic ocean. *J. Mar. Syst.* 103831 (2022) doi:10.1016/j.jmarsys.2022.103831.
16. Zhang, Y., Yu, W., Chen, X., Zhou, M. & Zhang, C. Evaluating the impacts of mesoscale eddies on abundance and distribution of neon flying squid in the Northwest Pacific Ocean. *Front. Mar. Sci.* **9**, (2022).
17. Ariza, A. *et al.* Global decline of pelagic fauna in a warmer ocean. *Nat. Clim. Change* **12**, 928–934 (2022).
18. Haris, K. *et al.* Sounding out life in the deep using acoustic data from ships of opportunity. *Sci. Data* **8**, 23 (2021).
19. AODN. Australian Ocean Data Network.
20. Habasque, J. *et al.* French PIRATA cruises: acoustic data. (2020) doi:10.17882/71379.
21. SEANOE. Sea Open Scientific Data Publication. (2021).
22. British Antarctic Survey - Natural Environment Research Council. Water column acoustic data collected in the Atlantic Ocean between 2006 and 2017. (2017).
23. BODC. British Oceanographic Data Centre.
24. PANGAEA. Data Publisher for Earth & Environmental Science. (2021).
25. IMARPE. Crucero de evaluación hidroacústica de recursos pelágicos BICs Humboldt, Olaya, SNP-2 y LP IMARPE IV 0110-11. (2004).

26. Conchon, A. & Cotté, C. THEMISTO cruises, RV Marion Dufresne. (2017).

27. MESOPP. Mesopelagic Southern Ocean Prey and Predators.

28. Irigoien, X. *et al.* The Simrad EK60 echosounder dataset from the Malaspina circumnavigation. *Sci. Data* **8**, 259 (2021).
