# Supplementary material for: A rare oasis effect for forage fauna in oceanic eddies at the global scale
Source: Nat Commun. 2024 Jun 6;15:4834. doi: 10.1038/s41467-024-49113-3 (PMC11156932; doi:10.1038/s41467-024-49113-3)
Supplement: Supplementary file 3 — Reporting Summary [file 41467_2024_49113_MOESM3_ESM.pdf]

nature portfolio

Corresponding author(s): Aurore Receveur
aurore.receveur@fondationbiodiversite.fr

Last updated by author(s): May 2, 2024

Reporting Summary

Nature Portfolio wishes to improve the reproducibility of the work that we publish. This form provides structure for consistency and transparency in reporting. For further information on Nature Portfolio policies, see our Editorial Policies and the Editorial Policy Checklist.

Statistics

For all statistical analyses, confirm that the following items are present in the figure legend, table legend, main text, or Methods section.

n/a	Confirmed	
☐	☒	The exact sample size (*n*) for each experimental group/condition, given as a discrete number and unit of measurement
☐	☒	A statement on whether measurements were taken from distinct samples or whether the same sample was measured repeatedly
☐	☒	The statistical test(s) used AND whether they are one- or two-sided<br>*Only common tests should be described solely by name; describe more complex techniques in the Methods section.*
☐	☒	A description of all covariates tested
☐	☒	A description of any assumptions or corrections, such as tests of normality and adjustment for multiple comparisons
☐	☒	A full description of the statistical parameters including central tendency (e.g. means) or other basic estimates (e.g. regression coefficient) AND variation (e.g. standard deviation) or associated estimates of uncertainty (e.g. confidence intervals)
☐	☒	For null hypothesis testing, the test statistic (e.g. *F*, *t*, *r*) with confidence intervals, effect sizes, degrees of freedom and *P* value noted<br>*Give P values as exact values whenever suitable.*
☒	☐	For Bayesian analysis, information on the choice of priors and Markov chain Monte Carlo settings
☒	☐	For hierarchical and complex designs, identification of the appropriate level for tests and full reporting of outcomes
☒	☐	Estimates of effect sizes (e.g. Cohen's *d*, Pearson's *r*), indicating how they were calculated

Our web collection on statistics for biologists contains articles on many of the points above.

Software and code

Policy information about availability of computer code

Data collection	Data were downloaded and read from public repositories in internet data portals using custom software implemented in Python 3.8.
Data analysis	Data were all analysed with the R software, version 4.2.1. All analysis packages used are open-source and analysis procedures are provided in the manuscript. All the codes necessary to reporduce the analysis and the Figures are publicly avilable here: https://github.com/auroreRECE/eddy_micronecton/tree/main

For manuscripts utilizing custom algorithms or software that are central to the research but not yet described in published literature, software must be made available to editors and reviewers. We strongly encourage code deposition in a community repository (e.g. GitHub). See the Nature Portfolio guidelines for submitting code & software for further information.

Data

Policy information about availability of data

All manuscripts must include a data availability statement. This statement should provide the following information, where applicable:

- Accession codes, unique identifiers, or web links for publicly available datasets
- A description of any restrictions on data availability
- For clinical datasets or third party data, please ensure that the statement adheres to our policy

All data used in the present study are publicly available, excepted three acoustic surveys.
Eddy trajectories and characteristics are available on the AVISO data page (https://www.aviso.altimetry.fr/en/data/products/value-added-products/global-

mesoscale-eddy-trajectory-product.html)
Acoustic data can be accessed through diverse internet repositories indicated in Supplementary Table S2. Raw acoustic data from the Malaspina circumnavigation expedition were processed using the open-source software Matecho v.6.7 following the standard procedures detailed in 26. The rest of the acoustic repositories were already available as processed data. The three Mozambique channel surveys are available upon request.
SST and chlorophyll data were downloaded through Copernicus website. The products ID are: “SST_GLO_SST_L4_REP_OBSERVATIONS_010_024” (SST) and “OCEANCOLOUR_GLO_BGC_L4_MY_009_104” (chlorophyll).
Source data are provided with this paper.

Research involving human participants, their data, or biological material

Policy information about studies with human participants or human data. See also policy information about sex, gender (identity/presentation), and sexual orientation and race, ethnicity and racism.

Reporting on sex and gender	N/A
Reporting on race, ethnicity, or other socially relevant groupings	N/A
Population characteristics	N/A
Recruitment	N/A
Ethics oversight	N/A

Note that full information on the approval of the study protocol must also be provided in the manuscript.

Field-specific reporting

Please select the one below that is the best fit for your research. If you are not sure, read the appropriate sections before making your selection.

☐ Life sciences ☐ Behavioural & social sciences ☒ Ecological, evolutionary & environmental sciences

For a reference copy of the document with all sections, see nature.com/documents/nr-reporting-summary-flat.pdf

Ecological, evolutionary & environmental sciences study design

All studies must disclose on these points even when the disclosure is negative.

Study description	We compiled the most extensive database of underwater sonar data to study the influence of mesoscale oceanic eddies on the forage fauna. we characterized the response of the sonar-detected forage fauna to 999 eddies across the oceans, the largest number of satellite-detected eddies ever sampled by sonar acoustic.<br>While we show that these eddies affect surface temperatures and phytoplankton, the impacts on the forage fauna are limited to 13% of cases. Only a few eddies, characterized by a strong amplitude and a high-water-mass-trapping ability aggregate forage fauna.<br>The study implies quantitative data. In each sample eddy (n=999), the outside values were compared to the inside values with two-sided Wilcoxon distribution test at 95% confidence level.
Research sample	Open-ocean pelagic forage fauna detected with ship-born acoustic echosounders from 20 to 750 m depth. Mostly mid-trophic level organisms such as small fish, crustaceans and diverse gelatinous taxa.<br>The different data sources are described above in the data availability statement. The dataset is composed of 999 sampled eddies.
Sampling strategy	We first compiled the largest acoustic database at 38 kHz available to date in public repositories. This dataset is composed of 286,919 vertical acoustic profiles.<br>Then, we overlapped the acoustic database with the sattelite-detected eddies. We extracted the eddies from the largest global atlas. In this atlas, the eddies are detected and tracked based on the Sea Surface Level Anomaly.<br>By overlaping the eddies and the acoustics, we sampled 999 eddies spread over the globe with 117,833 vertical acoustic profiles. The previous studies on this topic never overpassed 15.
Data collection	The data were collected worlwide by research vessels from different reasarch institute and ships of opportunity. The main contributors are:<br>CSIRO, Australia<br>French Research Institute for the Development (IRD) France<br>BAS, United Kingdom<br>IMARPE, Peru<br>UPMC, France<br>CSIC, Spain<br>Institute of Marine Research (IMR, Norway)<br>More details are given in Supplementary Table S2.

Timing and spatial scale	Global acoustic database collected between 2001 to 2020, 1 and 1.8 km horizontal and 10 m vertical resolution. The years from 2002 to do not test for any temporal aspect, this does not change any results.
Data exclusions	Only acoustic profiles with missing or corrupted data between 20 and 750 m depth, collected during twilight, or where the seabed was above 1000 m depth were excluded.<br>Eddies with a lifespan inferior to 14 days were excluded to ensure that sampled eddies where enough coherent structure.
Reproducibility	For the sake or reproducibility, all data analyzed in the present study are publicly available, analyses were thoroughly described in the manuscript, they all were conducted with open-source tools, and all the codes are publicly available.
Randomization	All attempts to repeat the data analysis were successful.
Blinding	Our study did not require blinding because it did not involve experiments. We repeat the statistical analysis in different wat, and by different personns, and the results always made consensus.

Did the study involve field work? ☐ Yes ☒ No

Reporting for specific materials, systems and methods

We require information from authors about some types of materials, experimental systems and methods used in many studies. Here, indicate whether each material, system or method listed is relevant to your study. If you are not sure if a list item applies to your research, read the appropriate section before selecting a response.

Materials & experimental systems

n/a	Involved in the study	
☒	☐	Antibodies
☒	☐	Eukaryotic cell lines
☒	☐	Palaeontology and archaeology
☒	☐	Animals and other organisms
☒	☐	Clinical data
☒	☐	Dual use research of concern
☒	☐	Plants

Methods

n/a	Involved in the study	
☒	☐	ChIP-seq
☒	☐	Flow cytometry
☒	☐	MRI-based neuroimaging

Plants

Seed stocks	N/A
Novel plant genotypes	N/A
Authentication	N/A
